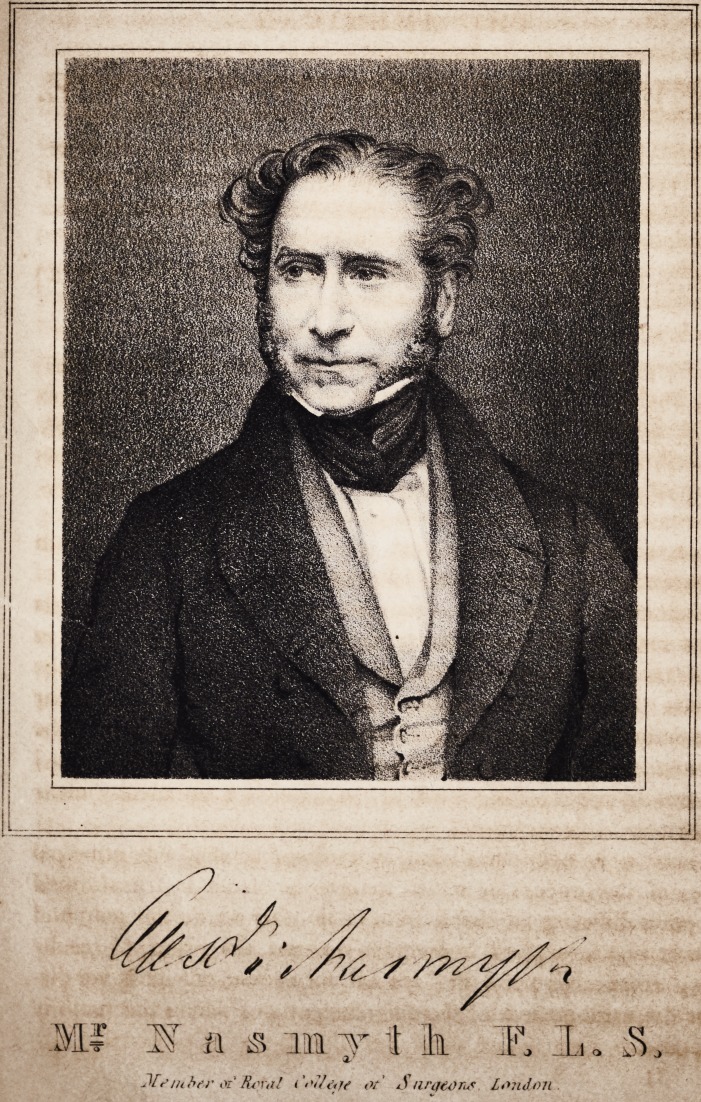# Memoir upon the Cellular Structure of the Teeth and Their Pulps; upon the Formation of the Ivory That Contains Them; and upon Some Points of Odontology

**Published:** 1843-06

**Authors:** Alexander Nasmyth

**Affiliations:** Member of the Royal College of Surgeons; London.


THE AMERICAN

### JOURNAL OF DENTAL SCIENCE.

VOL. III.] JUNE, 1843. [No. 4.

[EXTRACT from the Proceedings of the Academie des Sciences, Oct. 3, 1843.]

### ARTICLE I.

MEMOIR *upon the Cellular Structure of the Teeth and their Pulps; upon the Formation of the Ivory that contains them; and upon some points of Odontology.* By ALEXANDER NASMYTH, Member of the Royal College of Surgeons; LONDON. Translated from the French, for the American Journal of Dental Science.

THE structure of every part of a tooth, though differing in appearance, is the same and is subject to the same modes of development. An areolar tissue, the disposition of whose cells varies according to the parts, but whose existence is evident in all, in the enamel as well as the pulp, forms the net-work, the canvas. Such is the proposition which would necessarily appear to be deduced from the facts contained in the memoir I had the honour to submit, two years ago, to the Academy; facts which I was wrong in not setting forth, at the time, in a sufficiently clear manner.

I now now ask permission to review, briefly, the principal phases of the process by which the areolar tissue is transformed into parts differing so much from each other as do the pulp and enamel; and to explain, in a simple manner, the principal results of my researches upon the special forms under which we discover the arrangement of the different parts of which the tooth is composed.

The pulp is formed of two distinct tissues; the one vascular, is designed to supply the elements of nutrition and transformation; the one reticular has deposited in its cells the calcareous salts which transform the pulp into ivory. The first, or vascular consists of trunks which divide into numerous and delicate branches. These having arrived to near the surface of the pulp, bend themselves into anastomosing channels, double upon themselves, then re-unite in trunks which carry back the blood to the venous circulation. The branches of the vascular apparatus form what has been called the *intermediate system.*

Since I presented my memoir to the institution, in 1840, I have traced, with a facility which has never before been done, the last ramifications of these vessels, by a method of injection which I propose to lay before the Academy in a future communication. Several of the preparations which I now submit for examination, seen through a microscope, demonstrate all the beauty and extent of this special circulation. In the most elevated point of their passage, the walls of the intermediate capillary vessels are in immediate contact with the cells of the areolar tissue which surrounds them. Though the diameters of these capillaries are generally uniform, they nevertheless present some dilatations upon the injected pieces.

If we make an incision upon the tooth, passing at once through the ivory and the pulp, we will see that it is in the most elevated cells of the latter that the calcareous salts are deposited, which give to the portion of the tooth where that operation is accomplished, the hardness and the other physical characters to which it owes the name of ivory. Many of my preparations afford remarkable examples of that transformation. When we examine them with a magnifying power, four or five hundred times, the last cells of the pulp, we observe an arrangement altogether peculiar, and which makes them resemble the fibres of a dead and dried leaf.

If we examine a part of a tooth in which ossification is complete, it is impossible not to observe that the ivory has been really deposited in the niches formed for it by the pulp. Indeed, these preparations enable us to distinguish, by the semi-transparence of the calcareous salts, not only the walls of the cells which, being formed of animal matter, are less transparent than the saline parts, but even the corpuscles (nucleus) of each cell, which encrusted also with calcareous matter, offers remarkable differences, as the section of the tooth has been transverse or longitudinal. This arrangement has led me to explain, as an optical illusion, the mistake of observers, who, having distinguished, by the longitudinal section of the tooth, some lines less transparent or dark, have supposed that they had discovered little canals, while, in truth, the presence of these dark lines is but the result of the less transparency of the corpuscles of animal matter, which, in the longitudinal section, are arranged in series, or under the form of a chaplet. It is to this series of corpuscles that I have given the name of fibres, because they represent, in fact, what is distinguished by this name in other structures or tissues.

I will not reproduce here the proofs I have adduced in my memoir, in evidence of this explanation. There is one, however, which is so striking that I will mention it in a few words: if we treat with acids one of these preparations, where these pretended tubuli are found, which are supposed to be furrowed in the calcareous matter, after all this matter shall have been destroyed, we will yet see the black lines, but evidently produced by a series of corpuscles of animal matter.

Hence the ivory or bone is a portion of the pulp ossified, and in which the difference of transparency, or the elements which compose it, permits us to distinguish the walls of the cells and the corpuscles which each of these contain. Consequently the ivory, according to my investigation, comes under the province of the organic laws which Schwann first traced out with so much care and was afterwards followed by others. However, with due deference to the correctness of the views to which the German physiologist arrived, I think it proper to point out the difference which exists between the general declaration of Schwann, and the positive results to which I have arrived by minute researches upon this point of the science, to which Schwann never directed his special investigations. Although his work was published at the period when I addressed my first communication to the congress of Birmingham, I had no knowledge of it.

As to the mode of nutrition and ossification of ivory, judging from perfect injections which I made, no vessel was discovered to penetrate into that substance. I can account for these two processes by the exosmosis of a fluid carried by the vessels which are found in immediate contact with the walls of the cells.

I have made the same experiments and obtained like results, in the investigation of the enamel and of cement; and, in these various products, I have always discovered the same cellular organization as I did in the pulp and ivory, but with new modifications.

The arrangement of the cells of the ivory, the enamel and cement varies in different classes of animals, but is the same in each species. I shall speak of the direction of the fibres of the ivory which radiate near the surface, according to the class of animals. Hence the organization of the different parts of a tooth exhibits to the zoologist a new means of distinguishing the different classes of animals; and this means is not only applicable to those which are actually found upon the surface of the earth, but also to the species, the remains of which are only found in a fossil state. I have lately had occasion to investigate this method, and to discover its utility by an examination of collections of fossils, brought from America to England by Mr. Kock. Those fossils which resembled the organs of the mastadon had belonged to a single species. My friend, Professor Grant, having imagined that he discovered in them the remains of five different species, proposed to me to examine them by the method of which I have spoken, and, in fact, by the examination of the intimate organization of the teeth of these fossils, I arrived at the same conclusion. I have communicated the results of that investigation to the Geological Society of London, in the *Transactions* of which it will probably be published; and Professor Grant proposes to insert them in the work which he is preparing upon this important description of animals.

## Figures and Tables

**Figure f1:**